# Filling Teeth

**Published:** 1853-01

**Authors:** 


*Filling Teeth.* BY DR. CLARK.

DR. JAS. TAYLOR, DEAR SIR:—In your April No. of the Register, you were pleased to notice favorably some operations of mine. Also expressing a wish for some account of my mode of operating. I need not say that I felt flattered by so kind a notice, coming as it did, from one of the pioneers in our "noble profession." But I will say that I hold all I have as an operator in Dental Surgery, as not belonging more to me than to the profession, and it will give me sincere pleasure to comply with your request, if I may add to or stimulate the endeavor of any of my co-laborers to attain a single point more of excellence or facility in their operations.

In filling I use my gold in cylinders entirely, which are set up parallel to the walls of the cavity until the cavity is, *apparently* full. A round pointed instrument is then forced down between them and others inserted (decreasing in size) until it is LITERALLY full. These cylinders are made thus:

I take the small "pivot broach" (Watchmaker's) and break it off, say one eighth of an inch from the shoulder, this part of the blade is then reduced to a perfect point, preserving the broach cutting edge, as perfectly as possible, I fold the gold in strips as wide as I wish my cylinders long. To catch the end of the strips on this broach point, between the thumb and forefinger of the left hand, and to rotate the broach with the right will be obviously easy for any one to produce a perfectly even and smooth cylinder, of any desired size. The manner of introducing these is, of course, with tweezers or "filling forceps." I have used, with much satisfaction, "Taylor's

Filling Forceps," and also one pair of straight and one curved of the watchmaker's hair spring tweezers, reduced to the size of ordinary pin wire, at and near the points.

These cylinders are made of different density. The first and those placed against the walls of the cavity are rolled lighter than those introduced last, which are made quite hard, for obvious reasons. Some are made conical or slightly so, and introduced according to the shape of the cavity, with the base of the cone in or out, as the shape may require. Frail teeth, that I could not fill safely with the strip, roll, or pellet, I think I am able to fill usefully with cylinders, while great care must be taken not to fracture the tooth with the condenser, between the cylinders. A strong molar (to my knowledge) may be split open easily by too much force in adding the last (or key stone) cylinder. Advice or opinions are given to be considered and followed or not, as they may appear necessary or useful. With this view and with due defference to others, I will give my opinion, in regard to the last part of the operation *finishing.*

I do not think it necessary to produce the finest polish possible on the filling, but I do think it absolutely necessary to bestow as much labor on the tooth and orifice, when it has been cut or filed as will ensure a fine polish on the filling.

As a matter of taste too, I do not know any good reason why ladies and gentlemen should be so particular about the finish of an almost useless article of jewelry and not have some show of care in the polish of their "*mouth jewels.*" In this process, I employ cuting and polishing substances, for I have no confidence in those fillings that are trusted to the file and burnisher alone. By the way, more (otherwise) well filled teeth (especially if the gold is not the most dense) are ruined, in my opinion, by that burnisher, than almost any other mechanical cause.

If it is doubted, file down a filling just level, use a pretty round faced burnisher, with force, over the surface and orifice, then take a good lens and examine it. Look at it six months or a year after and you will find, in the fractured orifice, the precursors of ruin to your operation, if nor to the tooth. The advantages that I think may be claimed in favor of the cylinder fillings are these:

The filling is in the form of an arch, calculated to make up, as far as possible, for the loss by disease.

The gold, all lies parallel to the walls of the cavity, an obvious advantage.

Any one trying it will be forced to admit that he can harden and finish the ends of the cylinder (or edges of the foil) more perfectly than a surface of more irregular lamina.

In speaking of the cylinder I do not forget that foil is and may be put in, embracing the same points in principle. The name is nothing, I have only retained it as illustrating a principle.

*Fang Fillings.*—The preparation of the pulp cavity, by the removal of the dental pulp, I need not describe, only that I use the finest watchmaker's broach, untempered. Barbs are easily cut with any cutting instrument. With these there is no difficulty, in all ordinary cases, in getting out the pulp neat and clean. The Yankees have a word that just expresses what will reward the patient, careful operator—he will soon get the "knack of it." When he has got it he will find the pulp reversed with the small end out first in most cases. The foil is easily introduced by cutting a strip of single leaf and winding (as in the case of the cylinder) to the very point of an untempered smooth broach, increasing the size back, in shape and size, to suit the shape of the pulp cavity, making it somewhat longer than needed. This broach is inserted into the fang, with the gold on it. Then, by grasping the gold lightly with a pair of tweezers, the broach may be withdrawn, leaving the gold in the fang. A piece of whalebone, nicely scraped down to the shape of the pulp cavity, is thrust up by the side of the gold, making room for another, as before, and so on until the fang is full. It is unnecessary to say that several broaches, thus prepared, are necessary, in commencing to fill a fang or fangs.

I cannot close this without doing the justice of gratefully acknowledging indebtedness to Dr. F. H. Badger, for the mode of performing this last operation, of introducing gold into the fang, and also an intimation of the principle of cylinder filling. How he made the cylinders I never knew till very recently. I now understand that he takes a strip folded, never less than half an inch wide, and puts the ends continually together, until it is nearly round, when he finishes it, by rolling it in the palms of his hands, and then he cuts off the length wanted for use.

I chose the other method, as I knew no other and thought that the best to make a perfect cylinder and easily shaped as I desired it.

I still think it such, and, having tried the other method lately, I found the cylinders less perfect and too much irregularly, hardened by the cutting of them to the proper length.

In my method, cylinders are easily made of conical shape, by folding the strip thicker on one end than the other. To me, that shape is essential. By rolling up a literal wire, cuting off to the length desired for use, that shape connot be produced.

*Queries.*—I have stated that a harder and more perfect surface can be obtained by the cylinder principle of filling.

All fluids we know are composed of globules; gold when melted is a fluid.

Do those globules flatten in the process of lamination and present, on the edge of the foil, a more tense resistance or compact grain than the side or surface of the lamina?

We understand that a cohesive union takes place between the lamina of gold brought in contact and pressure applied.

To fulfil the full conditions of this union, we understand that the air must be expelled perfectly. Does the cylinder filling offer any facilities to this expulsion of the air over an irregular twisted roll, a crumpled pellett, or ball, of irregular lamina?

